# Relationship Between Historical Lameness, Medication Usage, Surgery, and Exercise With Catastrophic Musculoskeletal Injury in Racehorses

**DOI:** 10.3389/fvets.2018.00217

**Published:** 2018-09-07

**Authors:** Peta L. Hitchens, Ashley E. Hill, Susan M. Stover

**Affiliations:** ^1^J.D. Wheat Veterinary Orthopedic Research Laboratory, School of Veterinary Medicine, University of California, Davis, Davis, CA, United States; ^2^Equine Centre, Melbourne Veterinary School, Faculty of Veterinary and Agricultural Sciences, University of Melbourne, Werribee, VIC, Australia; ^3^California Animal Health and Food Safety Laboratory System, University of California, Davis, Davis, CA, United States

**Keywords:** epidemiology, musculoskeletal injury, horse, equine, fatality

## Abstract

**Background:** The rate of catastrophic musculoskeletal injuries (CMI) in racehorses is high in the United States compared to other countries. Few modifiable risk factors related to lameness, medication, and surgery history have been identified.

**Objective:** To detect management factors that increase risk of CMI by comparing medical histories between horses that sustained, and horses that did not sustain, a CMI.

**Study design:** Case-control.

**Methods:** Racehorse necropsy data (May 2012-June 2013) were obtained through the California Horse Racing Board Postmortem Program. Attending veterinarians of Thoroughbreds (TB) and Quarter Horses (QH) that experienced CMI, and of three matched control horses, were invited to complete an online veterinary medical history survey. We investigated associations between CMI and lameness, medication, surgery, and exercise history using multivariable logistic regression.

**Results:** There were 146 TB (45 cases, 101 controls) and 17 QH (11 cases, 6 controls) surveys completed. TB cases were more likely to show signs of lameness within the 3 months prior to death compared to controls. A high proportion of both cases (64.3%) and controls (76.8%) were administered medications, but unraced TB case horses were more likely to have been administered systemic medications compared to those that previously raced. TB cases were more likely to have raced with greater intensity during their career, but had eased off in the month preceding CMI. For QHs, there was insufficient power to detect significant differences between cases and controls that showed signs of lameness, or that were administered medications. Surgery history was not associated with CMI.

**Main limitation:** Insufficient power to detect small effect sizes.

**Conclusions:** The study provides information that can be used to aid in identification of horses at high risk for catastrophic injury, and management factors that can be modified to reduce the risk for all horses.

## Introduction

Musculoskeletal injuries are the primary cause of death for racehorses (1–4), and in California, there are about two catastrophic musculoskeletal injuries (CMI) for every 1,000 race starts in both Thoroughbred (TB) (5–7), and Quarter Horse (QH) racing (3, 7). This incidence of CMI is higher than the incidence reported in other countries such as Australia and New Zealand (0.4/1,000 starts) [(2, 8)], and the United Kingdom (0.7/1,000 starts) (4). California has seen a reduction in the frequency of fatalities in recent years (9), with this reduction credited to the implementation of a Racing Safety Program that has included horseshoe restrictions, improvement of track surfaces, restrictions on injection of some medications (e.g., corticosteroids), and continuing education of trainers. Implementation of other factors related to the management of the horse should therefore also influence the incidence of such injuries.

Risk factors investigated for CMI in racehorses have been well reviewed (10–16). Factors associated with increased risk of CMI or fatality include horse and race-level characteristics such as horse age (4, 17–23), age at first start (21–23), horse sex (17, 19, 22, 24, 25), race distance (21–24, 26), type and condition of track surface (4, 20–24, 27–30), class of race (18, 22, 24), and number of race starters (18). Factors related to management or training and racing programs that are associated with CMI include prior racing history (21, 22, 25, 31–33), horse shoe characteristics (e.g., use of toe grabs) (25, 34), and lay-up history or extended intervals since last race (24, 25, 32). Additionally, musculoskeletal injury has been reported to be associated with abnormal prerace physical inspection findings (26, 35), previous number of injuries, being scratched or having been on the vet list (23), and with administration of non-steroidal anti-inflammatory drugs (NSAIDS) or corticosteroids (36, 37). Despite this long list of risk factors, factors related to history of lameness, administration of all types of medication, and surgery have yet to be identified.

To obtain information on these factors, attending veterinarians of horses that experienced a CMI, and of control horses (horses that competed in the same race or trained on the same day as their respective case but did not have a fatal injury), were invited to complete an online veterinary medical history survey. The aim of this survey was to identify information that can be used to aid in identification of horses at risk of catastrophic injury, and management factors that can be modified to reduce this risk in racehorses.

## Methods

### Study population

The target population were attending veterinarians of TB and QH racehorses that experienced a CMI during racing or training (case) and of three matched control horses, in California between May 1, 2012 and June 30, 2013.

Case horses were racehorses that were necropsied through the California Animal Health and Food Safety Laboratory System (CAHFS) for the California Horse Racing Board (CHRB) Postmortem Program. CAHFS conduct post-mortem examinations of every horse that has died or was euthanized at racing or training facilities under the jurisdiction of the CHRB. Three control racehorses were selected for each case. For cases that died as a result of an injury incurred during racing, controls were selected from unaffected horses that competed in the same race. For cases that died as a result of an injury incurred during training, controls were selected from unaffected horses that recorded an official timed workout on the same day and at the same racetrack as their respective case, and matched on breed, age (years), and sex (intact male, neutered male, female). Career race and officially timed workout reports for each case and control horse were obtained from a commercial database of official racing industry records (InCompass Solutions Inc., The Jockey Club, Lexington, KY, USA). From these records, high-speed exercise history variables were generated using custom software detailed elsewhere (38, 39). Data were limited to high-speed exercise performed to the date of death of the respective case and included variables associated with their career (from the first recorded event to death), including number of events (races and official timed works), time between events, intensity of events, and lay-up (≥60 days without an official recorded event).

### Survey

A three-part 48-question confidential online survey was designed in the open source software LimeSurvey (Supplementary File 1; LimeSurvey 2.0, 2013; www.limesurvey.org). The survey was piloted by official veterinarians prior to study commencement. Consent was obtained from the attending veterinarian (having the owners' authority to act as agents for the owner in relation to veterinary matters). The information was maintained in confidence under the relevant law, including, but not limited to, the “official information privilege” (California Evidence Code section 1040) and the “veterinary services privilege” (California Business and Professions Code section 4857). Closed-ended multiple-choice and categorical questions were primarily used in the three sections: lameness history, medication history, and surgery history. Open-ended text fields were included at the end of each section to obtain more detailed information.

The attending veterinarians of the case and control racehorses were invited to voluntarily complete the online veterinary medical history survey. The individual survey links were distributed via email to the California Horse Racing Board official veterinarians, who forwarded the email on to the relevant attending veterinarian of each racehorse. Reminders were issued by email to participants that did not respond within one month of the date of death of the case horse. The mean time taken to complete a survey, from opening the online survey to submitting the survey, was 7.4 (standard deviation 7.6) minutes.

### Statistical analysis

Survey responses were compared between cases and controls, stratified by racehorse breed. Logistic regression was used to assess differences between groups for the whole dataset for lameness, medication administration, and surgery history, as well as between TB cases and controls for exercise history variables. Because there were only 31 cases with complete matching to controls (21 cases with one matched control, eight cases with two matched controls, and two cases with three matched controls), we fitted univariable logistic regression models, adjusting for clustering of random-effects on the variable indicating matched cases and controls (i.e., matched set id). This method avoided substantial data loss. To ensure our findings held up to this method we also fitted the subset of matched data using univariable conditional logistic regression models. Where a number in a 2 × 2 contingency table cell was <10, univariable exact logistic regression was employed. Cases or controls were omitted from analysis of specific medications where the attending veterinarian noted that it was unknown whether administration of medication had taken place while under their care or otherwise. Study factors in univariable analysis that were *p* < 0.20 were entered into a multivariable logistic regression model, adjusting for clustering of matched cases and controls, and omitted in a backwards stepwise manner. Variables were retained in the model if *p* < 0.05 or if they modified the coefficients of other covariates by more than 10%. For continuous variables, violation of the linearity assumption in the logit was assessed for each of the study factors using the Box-Tidwell transformation test. Two-way interaction terms between biologically plausible study factors were assessed. Model diagnostics of the final model included the Hosmer-Lemeshow's goodness-of-fit test, the link test to identify model specification error, examination of tolerance (>0.1), and the variance inflation factor (VIF < 10). A *p* ≤ 0.05 was considered statistically significant for all tests. Odds ratios (OR) and their 95% confidence intervals (95% CI) are presented. All statistical analyses were conducted using Stata, version 14.2 (StataCorp, College Station, Tex, USA).

## Results

Of the 660 surveys distributed to veterinarians of cases (*n* = 165) and controls (*n* = 495), 31.8% (*n* = 210) of surveys were initiated, and 29.2% (*n* = 193) were completed in their entirety. The 17 unusable survey responses were incomplete or were from veterinarians that indicated that they were not the primary attending veterinarian for that horse. Of the 193 complete surveys, 84.5% (*n* = 163) were completed by attending veterinarians of cases that died due to a CMI or their respective controls in association with training, racing, or non-exercise. The remaining 30 surveys were non-musculoskeletal cases (*n* = 4) and controls matched to non-musculoskeletal cases (*n* = 26) that were removed from analysis. Nine surveys (three cases, six controls) were completed for TB racehorses that died as a result of an injury incurred when not exercising. The three cases included skull fracture as a result of a severe procaine reaction, proximal phalangeal fracture of unknown cause, and a case of chronic laminitis. Of the CMI cases and controls, 33.9% (56/165) responses were from the attending veterinarian of a case horse, and 21.6% (107/495) were from the attending veterinarian of control horses. Only CMI cases, and respective controls that died associated with exercise, have been included in the following results.

The attending veterinarians were asked how long the horse was under their care: 9.2% (*n* = 15/163) of horses were under the veterinarians care for <1 month, 36.2% (*n* = 59/163) for 1–6 months, 21.5% (*n* = 35/163) for 6–12 months, and 33.1% (*n* = 54/163) for more than 12 months. There were 146 surveys (45 cases, 101 controls) completed for Thoroughbreds and 17 surveys completed for Quarter Horses (11 cases, 6 controls).

### Racing or training

For TBs, 72 (25 cases, 47 controls) of the surveys were related to a CMI incurred by the case during racing, and 65 (17 cases, 48 controls) were related to a CMI incurred by the case during training. For QHs, all 17 (11 cases, 6 controls) of the surveys were related to a CMI incurred by the case during racing.

In the free text notes, 24.0% (6/25) of the attending veterinarians of cases and 14.9% (7/47) of controls in the TB racing sample; 17.6% (3/17) of cases and 2.1% (1/48) of controls in the TB training sample; and 0% (0/11) of the cases and 16.7% (1/6) of the controls in the QH racing sample, noted that the reason for missing or unknown information was that the horse had been claimed, shipped in, or changed trainers in the previous 12 months.

### Lameness

For both TBs and QHs there were no significant differences between cases and controls in proportion of lameness observed, although 37.8% (14/37) TB cases were lame in the 0–3 months prior to CMI compared to 23.3% (21/90) of controls (*p* = 0.115) (Tables 1, 2).

**Table 1:** Associations from univariable logistic regression models, adjusting for matching of cases and controls, between catastrophic musculoskeletal injury sustained during racing or training and lameness, medication and surgery histories of Thoroughbred racehorses in California, 2012–2013.

	**Cases (*****n*** = **42)**		**Controls (*****n*** = **95)**		**Odds ratio (95% CI)**	***p*-value**
	**Yes**	**No**	**Yes**	**No**		
Lameness						
0–3 months	14	23	21	69	2.00 (0.84, 4.74)	0.115
3–6 months	4	27	14	62	0.66 (0.14, 2.36)	0.702
6–12 months	7	12	22	41	1.09 (0.31, 3.52)	1.000
>12 months	5	7	10	36	2.52 (0.52, 11.79)	0.301
Anytime^*^	21	16	44	48	1.43 (0.65, 3.13)	0.368
IA or IT medications/injections	20	22	46	49	0.97 (0.47, 2.00)	0.931
Joint block						
Previous week	1	27	2	58	1.07 (0.02, 21.46)	1.000
Previous month	2	26	4	56	1.08 (0.09, 8.07)	1.000
>1 month	4	24	9	51	0.95 (0.19, 3.82)	1.000
Anytime	5	23	10	50	1.09 (0.26, 3.99)	1.000
Corticosteroids						
Previous week	4	26	8	64	1.23 (0.25, 5.08)	0.986
Previous month	8	22	20	52	0.95 (0.31, 2.68)	1.000
>1 month	9	21	19	53	1.19 (0.41, 3.33)	0.886
Anytime	15	15	28	44	1.57 (0.70, 3.54)	0.276
PSGAGS						
Previous week	5	19	15	46	0.81 (0.20, 2.79)	0.952
Previous month	6	18	17	44	0.86 (0.24, 2.79)	1.000
>1 month	3	21	13	48	0.53 (0.09, 2.23)	0.544
Anytime	18	6	19	42	0.74 (0.21, 2.36)	0.780
Hyaluronic acid (ha)						
Previous week	2	25	2	57	2.26 (0.16, 32.76)	0.745
Previous month	4	23	6	53	1.53 (0.29, 7.17)	0.770
>1 month	4	23	7	52	1.29 (0.25, 5.68)	0.948
Anytime	8	19	9	50	2.31 (0.67, 7.93)	0.210
IRAP						
Previous week	0	21	0	49	–	–
Previous month	0	21	0	49	–	–
>1 month	1	20	0	49	2.33 (0.06, +Inf)	0.600
Anytime	1	20	0	49	2.33 (0.06, +Inf)	0.600
Antibiotics						
Previous week	1	21	1	50	2.35 (0.03, 190.56)	1.000
Previous month	1	21	1	50	2.35 (0.03, 190.56)	1.000
>1 month	4	18	8	43	1.19 (0.23, 5.17)	1.000
Anytime	4	18	8	43	1.19 (0.23, 5.17)	1.000
Stem cells						
Previous week	0	21	1	47	2.29 (0.00, 89.14)	1.000
Previous month	0	21	1	47	2.29 (0.00, 89.14)	1.000
>1 month	0	21	0	48	–	–
Anytime	0	21	1	47	2.29 (0.00, 89.14)	1.000
Systemic medications	25	17	70	25	0.53 (0.24, 1.13)	0.100
Phenylbutazone (bute)						
Previous week	16	13	48	33	0.85 (0.37, 1.93)	0.691
Previous month	21	8	59	22	0.98 (0.44, 2.18)	0.958
>1 month	10	19	31	50	0.85 (0.35, 2.04)	0.714
Anytime	22	7	61	20	1.03 (0.35, 3.29)	1.000
Flunixin						
Previous week	9	13	23	39	1.17 (0.38, 3.52)	0.944
Previous month	11	11	34	28	0.82 (0.32, 2.11)	0.685
>1 month	4	18	21	41	0.44 (0.10, 1.57)	0.264
Anytime	12	10	35	27	0.93 (0.36, 2.35)	0.871
Ketoprofen						
Previous week	6	12	10	38	1.88 (0.46, 7.27)	0.457
Previous month	7	11	15	33	1.39 (0.38, 4.91)	0.760
>1 month	1	17	5	43	0.51 (0.01, 5.07)	0.949
Anytime	8	10	17	31	1.45 (0.41, 5.01)	0.692
Methocarbamol						
Previous week	5	13	12	41	1.31 (0.30, 5.01)	0.882
Previous month	6	12	19	34	0.90 (0.24, 3.11)	1.000
>1 month	2	16	17	36	0.27 (0.03, 1.36)	0.144
Anytime	6	12	22	31	0.71 (0.19, 2.43)	0.746
bute + flunixin	23	7	63	20	1.04 (0.36, 3.31)	1.000
bute + flunixin + ketoprofen	23	7	63	20	1.04 (0.36, 3.31)	1.000
psgags + ha	12	16	23	43	1.40 (0.57, 3.43)	0.459
steroids + psgags	19	12	36	38	1.67 (0.75, 3.70)	0.205
steroids + ha	15	16	29	44	1.42 (0.64, 3.15)	0.386
steroids + psgags + ha	19	13	37	38	1.50 (0.69, 3.25)	0.303
Total medications	27	15	73	22	0.54 (0.24, 1.25)	0.151
Tendon/ligament injections	3	39	6	89	1.14 (0.18, 5.67)	1.000
Diagnostic block	1	23	5	61	0.53 (0.01, 5.15)	0.981
Platelet rich plasma	0	24	0	62	–	–
IRAP	0	24	0	62	–	–
Stem cells	0	24	0	62	–	–
Other	2	24	1	53	4.33 (0.22, 265.36)	0.491
Surgeries	3	27	7	78	1.24 (0.19, 5.90)	1.000
Orthopaedic	3	27	1	78	8.46 (0.65, 460.60)	0.125
Non-orthopaedic	1	27	6	78	0.48 (0.01, 4.28)	0.878

*Reference category for interpretation of the Odds Ratio is absence of lameness, medication, or surgery*.

* *Anytime represents a positive answer to at least one of 0–3, 3–6, 6–12, or > 12 months; or previous week, previous month, or > 1 month categories. Where at least one cell contains a value <10, exact logistic regression odds ratios and p-values have been reported. Medications note: Other systemic medications reported include: Furosemide, DMSO, Osteoform, Vetalog, Ventipulmin, Tranq, Ace, Gastroguard*.

**Table 2:** Associations from univariable exact logistic regression models between catastrophic musculoskeletal injury sustained during racing and lameness, medication and surgery histories of Quarter Horses in California, 2012–2013.

	**Cases (*****n*** = **11)**		**Controls (*****n*** = **6)**		**Odds ratio (95% CI)**	***p*-value**
	**Yes**	**No**	**Yes**	**No**		
Lameness						
0–3 months	2	8	2	4	0.52 (0.03, 9.78)	0.604
3–6 months	0	7	1	5	0.86 (0.00, 33.43)	0.462
6–12 months	1	4	0	5	1.00 (0.03, +Inf)	1.000
>12 months	0	2	0	3	–	–
Anytime^*^	2	8	2	4	0.52 (0.03, 9.78)	0.604
IA or IT medications	7	4	5	1	0.37 (0.01, 5.52)	0.600
Joint block	0	6	1	3	0.67 (0.00, 26.00)	0.400
Corticosteriods	7	3	5	1	0.49 (0.01, 8.45)	1.000
PSGAGS	2	6	1	2	0.69 (0.02, 58.78)	1.000
Hyaluronic acid	1	6	2	1	0.12 (0.00, 4.05)	0.183
IRAP	0	6	0	3	–	–
Antibiotics	1	6	0	1	0.43 (0.01, +Inf)	1.000
Stem cells	0	6	0	3	–	
Systemic medications	8	3	6	0	0.41 (0.00, 4.43)	0.515
Phenylbutazone	7	2	5	1	0.72 (0.01, 17.65)	1.000
Flunixin	5	2	4	0	0.66 (0.00, 9.58)	0.491
Ketoprofen	6	2	3	1	2.00 (0.01, 27.88)	1.000
Methocarbamol	0	3	0	2	–	–
Tendon/ligament injections	0	11	0	6	–	–
Total medications	8	3	6	0	0.41 (0.00, 4.43)	0.515
Surgeries	1	6	0	3	0.43 (0.01, +Inf)	1.000

*Reference category for interpretation of the Odds Ratio is absence of lameness, medication, or surgery*.

* *Anytime represents a positive answer to at least one of 0–3, 3–6, 6–12, or > 12 months. Due to the low numbers, time of medication administration was not stratified as per Table 1*.

In the conditional logistic regression model using only the subset of matched cases and controls, cases had 4.34 (*n* = 58; 95% CI 1.16, 16.26; *p* = 0.029) greater odds of showing signs of lameness at any time compared to their matched controls, most evident for TB cases that showed signs of lameness within the three months prior to death. The matched subset of TB cases that died during training had 5.67 (*n* = 37; 95% CI 1.16, 27.70; *p* = 0.032) greater odds of being lame at any time compared to their matched controls; again, most evident for TB training cases that showed signs of lameness within the three months prior to death.

Most poor performance or lameness problems were localized to the forelimb (Supplementary Table 1). There was no significant difference in proportion of cases or controls with either hindlimb or forelimb lameness' (*p* = 0.705). For TB cases, 47.6% (10/21) were localized to a bone, 52.4% (11/21) to a joint, 19.0% (4/21) to a tendon or ligament, and one was a severe laceration (two cases had multiple affected structures). For TB controls, 47.7% (21/44) were localized to a bone, 34.1% (15/44) to a joint, 13.6% (6/44) to a tendon or ligament, 2.3% (1/44) to a muscle, and one each being a hoof abscess and greater trochanteric bursa (three cases had multiple affected structures). For QH cases that had lameness problems localized to a structure, both cases had multiple affected structures with 100% (2/2) localized to both a bone and to a joint, tendon, or ligament. For QH controls, 100% (2/2) were localized to a joint.

A definitive diagnosis for lameness was reached for 47.6% (10/21) of the lame TB cases and 68.2% (30/44) of the lame TB controls, and 50.0% (1/2) of the lame QH cases and 50.0% (1/2) of the lame QH controls. Procedures used to diagnose the condition included clinical evaluation (TB *n* = 18), diagnostic analgesia (TB *n* = 7), radiography (TB *n* = 19), nuclear scintigraphy (TB *n* = 5), and ultrasonography (TB *n* = 1). Other diagnostic procedures included blood work and alkaline phosphatase test. No procedures or treatments were reported for the QHs that showed signs of poor performance or lameness.

Of the TBs, 85.7% (18/21) cases and 97.7% (43/44) controls were treated for their diagnosed condition including: 66.7% (14/21) cases and 52.3% (23/44) controls administered medications, 4.8% (1/21) cases and 4.5% (2/44) controls underwent surgery, 0% (0/21) cases and 13.6% (6/44) controls underwent ESWT (shockwave), 4.8% (1/21) cases and 11.4% (5/44) controls underwent physical therapy, and 52.4% (11/21) cases and 43.2% (19/44) controls were sent to layup.

For both cases and controls, percentage of career in layup was associated with an increased likelihood of lameness at any time (OR 1.02; 95% 1.01, 1.03; *p* = 0.024) compared to TBs that were not lame. Non-lame TBs had a mean of 19.2% (±21.9%) of their career in layup compared to lame TBs with 30.2% (±25.7%). To assess whether information on lay-up given in the survey agreed with information obtained from the horse's high-speed exercise history we conducted logistic regression with indication of layup in the survey as the outcome. Indication of layup in the survey was associated with greater mean weeks in layup (OR 1.03; 95% CI 1.01, 1.06; *p* = 0.009) and a greater percentage of their career in layup (OR 1.03; 95% 1.01, 1.05; *p* < 0.001), derived from the career race and officially timed workout reports.

Of the TBs treated for poor performance or lameness, 42.9% (9/21) of cases and 54.5% (24/44) of controls remained in training with no disruption (*p* = 0.538), 28.6% (6/21) of cases and 43.2% (19/44) of controls returned to racing and performed as they had previously (*p* = 0.392), and 4.8% (1/21) of cases and 9.1% (4/44) controls returned to racing with worse performance (*p* = 0.953). This question did not apply to horses that were not lame at any time.

### Medication

There was no significant difference in proportions between TB cases and controls administered any medication, with 64.3% (27/42) of cases and 76.8% (73/95) of controls being administered any medication: 47.6% (20/42) of cases and 48.4% (46/95) of controls administered intra-articular or intrathecal injections, 59.5% (25/42) of cases and 73.7% (70/95) of controls administered systemic medication, and 7.1% (3/42) of cases and 6.3% (6/95) of controls administered tendon or ligament injections.

There were no significant differences between TB cases and controls administered specific medications, with the exception that TB training-related cases (38.5% or 5/13) were more likely to have been administered hyaluronic acid injections compared to controls (10.0% or 3/30) (OR 5.63; 95% CI 1.05, 30.26; *p* = 0.044). The effect of combinations of medications were also investigated for phenylbutazone (bute) and flunixin; bute, flunixin, and ketoprofen; polysulfated glycosaminoglycan (PSGAGs) and hyaluronic acid (HA); corticosteroids and PSGAGs; corticosteroids and HA; and corticosteroids, PSGAGs and HA. TB cases were found to have greater odds of being administered these combinations of medications compared to controls, but these results did not reach statistical significance (Table 1).

No significant difference in proportions of QH cases and controls that were administered medication was found, with 72.7% (8/11) of cases and 100% (6/6) of controls being administered any medication: 63.6% (7/11) of cases and 83.3% (5/6) of controls administered intra-articular or intrathecal injection, 72.7% (8/11) of cases and 100% (6/6) of controls administered systemic medication, and none of the cases or controls administered tendon or ligament injection (Table 2).

### Surgery

Eleven racehorses had a reported surgery (TB three cases, seven controls; QH one case). No significant difference was found in proportions between TB or QH cases and controls that underwent surgery, though a greater proportion of TB cases had orthopedic surgery compared to controls (Tables 1, 2).

Three TB cases had orthopedic fixation surgery within 12 months prior to death (two screw fixations, one both screw and plate fixation). These cases also had one tie back surgery, and two arthroscopies. One TB control underwent proximal phalangeal fragment removal; the other six TB controls underwent primarily non-orthopedic surgeries (two castrations, colic surgery, caslick, eye enucleation, and sequestrum removal). The one QH case underwent orthopedic surgery involving both screw and plate fixation to right shoulder, and non-orthopedic soft tissue surgery 0–3 months prior to death.

### High-speed exercise history

Univariable associations between CMI and high-speed exercise history variables for Thoroughbred racehorses are presented in Table 3. Case horses had greater career races and race distance, higher frequency of races per year, less time between races, greater time between works, lower work and event distances and more time between events in periods without layup, and had lower training and racing exercise intensity before death than control horses.

**Table 3:** Associations from univariable logistic regression models, adjusting for matching of cases and controls, between catastrophic musculoskeletal injury and exercise history variables for Thoroughbred racehorses in California, 2012–2013.

	**Cases (*****n*** = **42)**		**Controls (*****n*** = **95)**		**Odds ratio (95% CI)**	***p*-value**
	***n***	**Median (IQR)**	***n***	**Median (IQR)**		
Horse age (d)	42	1410 (1133, 1770)	95	1375 (1119, 1729)	1.00 (1.00, 1.00)	0.398
Horse sex						
Male gelding	14	**–**	37	**–**	Reference	
Male entire	13	**–**	22	**–**	1.56 (0.69, 3.53)	0.285
Female	15	**–**	36	**–**	1.10 (0.55, 2.20)	0.785
Career						
Raced						
Previously raced	35	**–**	79	**–**	Reference	
Unraced	7	**–**	16	**–**	0.99 (0.42, 2.32)	0.977
Races (No.)	42	11 (4, 17)	95	6 (2, 13)	1.41 (1.09, 1.83)^a^	**0.010**
Works (No.)	42	27 (16, 43)	95	24 (13, 43)	1.08 (0.84, 1.39)^a^	0.555
Events (No.)	42	39.5 (23, 63)	95	29 (15, 56)	1.13 (0.89, 1.45)^a^	0.313
Race distance (f)	42	63.25 (26.5, 111.5)	95	39.5 (9, 84.5)	1.31 (1.03, 1.68)	**0.029**
Work distance (f)	42	111.5 (69, 172)	95	101 (51, 188)	1.05 (0.84, 1.32)	0.640
Total (event) distance (f)	42	174.75 (105.5, 318.5)	95	139 (65, 267)	1.09 (0.89, 1.35)	0.407
Mean race distance (f)	35	6.13 (5.43, 6.70)	79	6.50 (5.75, 7.45)	0.73 (0.51, 1.04)	0.077
Mean work distance (f)	41	4.14 (3.80, 4.33)	95	4.18 (3.71, 4.47)	0.89 (0.52, 1.50)	0.652
Mean event distance (f)	41	4.67 (4.29, 4.88)	95	4.67 (4.00, 5.03)	0.92 (0.65, 1.31)	0.657
Career length (d)	42	532 (212, 876)	95	487 (192, 808)	1.14 (0.93, 1.40)	0.218
Active career length (d)	42	425 (170, 678)	95	117 (301, 531)	1.15 (0.92, 1.44)^a^	0.212
Layup						
Number of layups	42	1 (0, 2)	95	1 (0, 2)	1.06 (0.72, 1.56)	0.756
Total layup time (d)	42	144 (0, 317)	95	130 (0, 277)	1.00 (1.00, 1.00)	0.801
Mean layup time (d)	42	125.5 (0, 187.5)	95	114 (0, 185)	1.00 (1.00, 1.00)	0.745
Percentage of career in layup	42	26.51 (0, 37.54)	95	23.27 (0, 40.41)	1.00 (0.98, 1.01)	0.811
Time since last layup (d)	42	194.5 (116, 372)	95	136 (75, 232)	1.22 (0.96, 1.54)^a^	0.102
Events since last layup (No.)	42	22 (14, 37)	95	15 (10, 26)	1.01 (0.99, 1.02)	0.236
Rate						
Career						
Races per year	42	6.15 (4.23, 9.82)	95	4.93 (2.25, 8.51)	2.03 (1.25, 3.31)^a^	**0.004**
Works per year	42	17.96 (13.11, 33.57)	95	23.69 (7.46, 32.68)	0.76 (0.51, 1.12)^a^	0.161
Events per year	42	26.06 (19.71, 41.48)	95	29.58 (23.79, 40.56)	0.79 (0.50, 1.25)^a^	0.314
Distance raced (f/mo)	42	3.12 (2.25, 5.09)	95	2.39 (1.19, 4.33)	1.10 (0.96, 1.27)	0.167
Distance worked (f/mo)	42	6.37 (4.40, 10.90)	95	8.40 (6.12, 11.56)	0.93 (0.85, 1.01)	0.081
Distance events (f/mo)	42	10.45 (7.50, 15.08)	95	11.75 (8.67, 15.56)	0.96 (0.90, 1.02)	0.194
Time between races (d)	34	38.86 (57.33, 78.47)	72	69.77 (42.96, 111.49)	0.99 (0.98, 1.00)	**0.011**
Time between works (d)	40	20.75 (11.58, 29.10)	93	15.83 (11.73, 21.27)	1.03 (1.00, 1.06)	**0.025**
Time between events (d)	40	14.29 (9.09, 19.05)	94	12.58 (9.73, 15.42)	1.02 (0.99, 1.06)	0.245
Active						
Races per year	42	9.39 (5.93, 11.23)	95	7.47 (3.82, 10.43)	1.97 (1.09, 3.58)^a^	**0.025**
Works per year	42	29.87 (21.53, 39.60)	95	34.88 (27.71, 43.34)	0.62 (0.37, 1.05)^a^	0.078
Events per year	42	37.65 (32.53, 47.20)	95	42.69 (36.73, 48.88)	0.48 (0.19, 1.22)^a^	0.122
Distance raced (f/mo)	42	4.57 (3.19, 5.77)	95	3.70 (2.09, 5.81)	1.04 (0.93, 1.15)	0.503
Distance worked (f/mo)	42	9.98 (7.37, 12.18)	95	12.01 (8.99, 14.81)	0.89 (0.82, 0.97)	**0.010**
Distance events (f/mo)	42	14.71 (11.97, 16.85)	95	16.24 (13.46, 18.86)	0.90 (0.83, 0.97)	**0.006**
Time between races (d)	34	40.99 (32.62, 49.63)	72	46.66 (34.84, 76.90)	0.99 (0.97, 1.00)	0.061
Time between works (d)	40	12.60 (10.30, 16.87)	93	10.93 (9.00, 13.79)	1.06 (0.99, 1.14)	0.078
Time between events (d)	40	10.16 (8.18, 11.45)	94	8.90 (7.90, 10.48)	1.16 (1.01, 1.32)	**0.035**
Exercise intensity (slope)						
Slope at start	34	12.69 (8.78, 16.62)	86	12.79 (9.73, 15.83)	0.99 (0.91, 1.08)	0.839
Slope at date of death	39	12.71 (8.35, 16.42)	92	16.62 (13.37, 20.66)	0.87 (0.82, 0.92)	<**0.001**
Slope after last layup	24	14.95 (8.93, 16.90)	60	16.84 (13.13, 19.24)	0.89 (0.81, 0.98)	**0.014**
Recent cumulative distances						
1 month (f)	42	14 (7.5, 19.5)	95	19 (14, 24)	0.91 (0.87, 0.95)	<**0.001**
2 months (f)	42	26 (15, 34)	95	33.5 (19.5, 40.5)	0.96 (0.94, 0.99)	**0.008**
4 months (f)	42	57.25 (27.5, 71.5)	95	53 (30, 72)	1.00 (0.99, 1.01)	0.781
6 months (f)	42	70.50 (31.5, 103)	95	65 (42, 102)	1.00 (0.99, 1.01)	0.666
8 months (f)	42	92.75 (45.5, 119)	95	75.5 (43, 114)	1.00 (1.00, 1.01)	0.570
10 months (f)	42	108.5 (48.5, 144)	95	86 (48, 125)	1.00 (1.00, 1.01)	0.668
12 months (f)	42	109.25 (48.5, 159.5)	95	88.5 (52, 149)	1.00 (1.00, 1.01)	0.796
Month 2 (f)	42	12.75 (4.5, 16.5)	95	13.5 (6.5, 18)	0.99 (0.94, 1.03)	0.557
Months 3 and 4 (f)	42	31 (12.5, 39)	95	20 (7, 35)	1.02 (1.00, 1.04)	**0.044**
Months 5 and 6 (f)	42	24 (1, 31.5)	95	10 (0, 32)	1.01 (1.00, 1.03)	0.145
Month 1 minus month 2 (f)	42	0.00 (-3, 7.5)	95	5.5 (2, 11)	0.91 (0.85, 0.97)	**0.004**

*Median values presented are untransformed and based on the pooled unmatched data. d, day; mo, month; No., number; f, furlong*.

a *Odds ratios determined from transformed data using the log base 2*.

### Multivariable analysis

In a multivariable model for TB cases and controls (Table 4), horses that sustained CMI were more likely to have shown signs of lameness <3 months prior to death. For TB CMI, 37.8% (14/37) of cases showed signs of lameness in the three months prior to CMI compared to 23.3% (21/90) of controls. TB cases that had previously raced (60.0%; 21/35) were less likely to have been administered systemic medications compared to controls (81.0%; 64/79); but unraced horses (57.1%; 4/7) were more likely to have been administered systemic medications compared to their controls (37.5%; 6/16) (interaction effect *p* = 0.023). For horses that had previously raced, more races per year over a horse's race career was associated with increased odds of CMI for a doubling of races. Additionally, more races in months three and four combined, but fewer cumulative furlongs worked and raced one month prior to case death were associated with CMI. Due to the small sample size, a multivariable model for the QH dataset could not be fitted.

**Table 4:** Associations from multivariable logistic regression models, adjusting for matching of cases and controls, between catastrophic musculoskeletal injury sustained during racing or training and lameness, medication and surgery histories of Thoroughbred racehorses in California, 2012–2013.

	**Odds ratio (95% CI)**	***p*-value**
**Main effects**		
Lameness 0–3 months	2.94 (1.05, 8.21)	0.040
Systemic medication	0.35 (0.10, 1.25)	0.105
Career		
Raced		
Previously raced	Reference	
Unraced	8.30 (0.45, 152.99)	0.155
Races per year	3.14 (1.39, 7.08)^a^	0.006
Recent cumulative distances		
1 month (f)	0.84 (0.76, 0.93)	0.001
Months 3 and 4 (f)	1.07 (1.01, 1.13)	0.014
**Interaction effect**		
Systemic medication # Raced		
Systemic medication, unraced	28.69 (1.61, 512.88)	0.023

*No., number; f, furlong*.

a *Odds ratios determined from transformed data using the log base 2*.

## Discussion

Modifiable risk factors for CMI related to lameness, medication, surgery and exercise history of TB and QH racehorses in California were investigated. TB cases were more likely to show signs of lameness especially within three months prior to death, and they were more likely to have been administered hyaluronic acid injections compared to controls. A high proportion of both cases and controls were administered medications, but unraced TB cases were the most likely to have been administered systemic medications. TB cases were more likely to have raced with greater intensity during their whole career including 3–4 months prior, but had eased off in the month preceding CMI. Significant differences for lameness and for medication history were not identified between QH cases and controls; but the sample size was small. Associations with surgery history for both breeds were not apparent.

TB cases were more likely to show signs of lameness within the three months prior to their death. Fewer furlongs raced and worked the two months prior to CMI for cases may have been an indication that case horses were unable to maintain high exercise intensity without developing lameness prior to a race. Reports of the association between lameness and racing are conflicting. Musculoskeletal conditions have been reported as the primary reason for not starting in a race (40, 41), and of days lost to training in the UK (41, 42), but in Germany the inability to train at high-intensity did not predict racing soundness, race starts or subsequent performance (43). Racehorses that have shown signs of lameness in the months preceding their fatal injury are likely to have a pre-existing condition. Such pre-existing conditions, stress fractures and/or pathology have been demonstrated in a high proportion of racehorses at postmortem (7, 44–47). We did not, however, have information on the severity of lameness, or on more specific lameness timeframes closer to the CMI occurring. This more detailed information may assist in differentiating these high-risk horses from others more readily.

A high proportion (73%) of all study horses were administered medication. Difficulties arise in determining whether medication is a risk factor for CMI due to such a high exposure amongst both cases and controls. However, we did find that TB cases that had previously raced were less likely to have been administered systemic medications, likely due to the restrictions of medicating close to race-day. Conversely, unraced case horses were more likely to have been administered systemic medications, possibly for the opposing reason. Although conducted more than three decades ago, Dirikolu et al. (36) also observed a high proportion of horses, in both injured and non-injured groups, with detectable levels of the NSAIDs phenylbutazone (83%) and flunixin (69%). Further, they found plasma concentrations of these NSAIDs were greater in injured horses when compared to a randomly selected horse in the same race (36). In this present study, associations with specific medications were not apparent, with the exception that cases that died as a result of a training-related incident had more than five times greater odds of being administered hyaluronic acid injections compared to controls. Although we found no significant differences between CMI cases and controls and use of corticosteroids, in an Australian study, Whitton et al. (37) reported a higher incidence of musculoskeletal injury (defined as a limb injury that resulted in the horse not racing for at least 6 months, or being retired) in horses administered local corticosteroid injections at any time compared to those that were not injected. Further, in their study horses administered two or more injections or with autologous conditioned serum had the highest incidence of musculoskeletal injury. The majority of musculoskeletal injuries (84%) occurred within two months of the last local corticosteroid injection. The addition of hyaluronic acid to corticosteroid injection did not significantly decrease incidence of musculoskeletal injury (37).

Thoroughbred cases had eased off racing one and two months preceding CMI. A study piloted by Cohen et al. (33) showed that horses that sustain musculoskeletal injuries during a race tend to have accumulated significantly fewer furlongs over the previous month or two months than horses that are not injured in the same race. By contrast, a study by Estberg et al. (17) found that horses that had accumulated more furlongs in the two months before a race were more likely to suffer a fatal injury during that race than horses that had accumulated fewer furlongs. Cohen et al. (33) speculated that the differences between their study in Kentucky and the study by Estberg et al. (17) in California could be due to differences between the populations, race surface, training or racing practices, or high-speed variables. Though our study was also from California, we showed similarities with that of Cohen et al. (33), and we similarly hypothesize that the lack of recent exercise may have been due to the development of pathology that limited the cases capacity for high-intensity exercise; or because lack of recent high-intensity exercise resulted in greater porosity and decreased bone density, as has been demonstrated in studies investigating fracture of the condyles of the third metacarpal bone (48, 49).

There are limitations that should be considered when interpreting the findings from this survey. The survey response rate was 32%, with subsequent incomplete matching due to the lower response of attending veterinarians of controls. Only large effect sizes were detected because we had only 58% power to detect an OR of 2.0, but 93% power to detect an OR of 3.0 at an alpha level of 5%. An estimated 92 cases, with complete 1:3 matching are required to detect an OR of 2.0 with power of 80%, assuming a prevalence of exposure among controls of 50%. The survey was initially planned to run for another complete year which, at the observed response rate, would have reached this target. Both missing information from completed surveys, misreporting, or non-response may be a source of bias. There is further potential risk of intentional censoring of medication history, or medication being administered without the knowledge or consent of the attending veterinarian. A higher proportion of case veterinarians noted that the reason for missing or unknown information was that the horse had been claimed, shipped in or changed trainers in the previous 12 months (17% of cases compared to 9% controls). Ideally, complete veterinary records of each horse would be the gold standard for use in analysis, however until complete veterinary records of all horses are required for regulatory purposes, this approach is not feasible. To facilitate ongoing investigation of risk factors, as well as being able to identify racehorses at risk for injury, it is imperative that detailed veterinary records accompany horses as they move between stables or training facilities. Lastly, questions regarding lameness could have been more specific. Although signs of lameness within 3 months prior to CMI was a significant risk factor, we do not know how close to the point of CMI the horse was observed to be lame, or indeed the severity of the lameness. It is also possible that the horse may have been lame during a specified time period, but that it was not attended to or reported to the veterinarian. Future surveys should consider including more detailed lameness questions.

Confirmation of the findings from this study, particularly those related to medication history, will provide information that can be used to aid in identification of horses at high risk for catastrophic injury, and management factors that can be modified to reduce the risk for all racehorses. Horses that have been observed to be lame should be differentially diagnosed where possible so that introduction to high-intensity exercise is not attempted prematurely. Identification of lameness and/or indicators of lameness should be pursued in future studies.

## Author contributions

SS was responsible for obtaining funding. PH conducted the survey and collated and analyzed the data. All authors were involved in study concept and design, interpretation of the data, drafting the article, critical revision, and final approval.

### Conflict of interest statement

The authors declare that the research was conducted in the absence of any commercial or financial relationships that could be construed as a potential conflict of interest.

## Supplementary material

Supplementary Figure 1 A paper-based version of the online veterinary medical history survey.

Supplementary Table 1 Affected structures of racehorses with poor performance or lameness problems in the months prior to case death, stratified by racehorse breed.
