# Supplementary material for: Relationship Between Historical Lameness, Medication Usage, Surgery, and Exercise With Catastrophic Musculoskeletal Injury in Racehorses
Source: Front Vet Sci. 2018 Sep 7;5:217. doi: 10.3389/fvets.2018.00217 (PMC6137211; doi:10.3389/fvets.2018.00217)
Supplement: Supplementary Figure 1 — A paper-based version of the online veterinary medical history survey. [file Data_Sheet_1.PDF]

Equine Medical History Survey

This **CONFIDENTIAL** survey is part of the Racing Injury Prevention Program conducted by the JD Wheat Veterinary Orthopedic Research Laboratory, University of California, Davis. The questions within this form are designed to allow detection of factors that increase risk for catastrophic skeletal injury by comparing medical histories between horses that had, and horses that did not have, a catastrophic injury.

The data arising from this survey will be used to develop strategies that aim to prevent injuries to race horses.

There are three (3) sections of this form:

1) Lameness

2) Medication

3) Surgery

To date, the average time for completion is <u>9 minutes</u>. Please answer questions to the best of your ability.

<u>DISCLAIMER</u>

By completing the survey you are agreeing to participate in this research. Please click on the 'I agree to participate' button at the bottom right of this page to signify your consent and agreement to participate in this research project.

The information you provide will be maintained in confidence to the full extent consistent with the technology being used and relevant law, including, but not limited to, the “official information privilege” (California Evidence Code section 1040) and the “veterinary services privilege" (California Business and Professions Code section 4857). Your participation in this online survey involves risks similar to a person’s everyday use of the Internet.

Thank-you for your participation.

There are 48 questions in this survey

Introduction

1 Only answer this question if the following conditions are met:

2 Are you the primary attending veterinarian for the horse:

Please choose **only one** of the following:

- Yes
- No

3 How long has the horse been under your care?

Please choose **only one** of the following:

- < 1 month
- 1 - 6 months
- 6 - 12 months
- > 12 months

4 If you are not the primary attending veterinarian, please contact {INSERT}.

5 If you have only been treating this horse intermittently, if the horse switched veterinary practices in the last 12 months, or if there are periods in last 12 months where you (or your practice) were not the attending veterinarian, please describe the circumstances:

6 If more than one veterinary practice has attended to the horse's care in the previous 12 months, please reply to {INSERT} and we can arrange for a separate link to be sent to the additional veterinarian.

Lameness

7 Have you attended the horse for any poor performance or unsoundness problems in the previous:

Please choose the appropriate response for each item:

	0 - 3 months	3 - 6 months	6 - 12 months	> 12 months
**Yes**	○	○	○	○
**No**	○	○	○	○
**Unknown**	○	○	○	○

8 If for unsoundness, which limb(s) or structure(s) were affected:

	0 - 3 months	3 - 6 months	6 - 12 months	> 12 months
**Left forelimb**	☐	☐	☐	☐
**Right forelimb**	☐	☐	☐	☐
**Left hindlimb**	☐	☐	☐	☐
**Right hindlimb**	☐	☐	☐	☐
**Vertebrae/spine**	☐	☐	☐	☐

You can select multiple time periods and structures.

9 Was at least one of these injuries localized to a structure?

Please choose **only one** of the following:

○ Yes

○ No

10 Was at lease one lameness localized to a bone, joint, tendon, ligament or muscle?

Please choose **all** that apply:

☐ Bone
☐ Joint
☐ Tendon or Ligament
☐ Muscle
☐ Other:

11 **<u>BONE (if you checked Forelimb and Bone in question 8 and 10 above)</u>**

<u>Forelimb:</u>

Please choose **all** that apply:

- ☐ Scapula
- ☐ Humerus
- ☐ Radius/Ulna
- ☐ Carpal bone
- ☐ Cannon bone
- ☐ Long pastern (Proximal phalanx)
- ☐ Short pastern (Middle phalanx)
- ☐ Coffin bone (Distal phalanx)
- ☐ Navicular
- ☐ Lameness not localized
- ☐ Other (describe):

12 ***<u>If you selected 'Long pastern', and if you are referring to a fetlock chip, please go back and select 'Joint' and then 'Fetlock' when prompted.</u>***

13 **<u>BONE (if you checked Hindlimb and Bone in question 8 and 10 above)</u>**

<u>Hindlimb:</u>

Please choose **all** that apply:

- ☐ Pelvis
- ☐ Femur
- ☐ Tibia/Fibula
- ☐ Hock bone
- ☐ Cannon bone
- ☐ Proximal sesamoid bone
- ☐ Long pastern (proximal phalanx)
- ☐ Short pastern (middle phalanx)
- ☐ Coffin bone (distal phalanx)
- ☐ Navicular
- ☐ Lameness not localized
- ☐ Other (describe):

14 ***If you selected 'Long pastern', and if you are referring to a fetlock chip, please go back and select 'Joint' and then 'Fetlock' when prompted.***

15 BONE (if you checked Spine and Bone in question 8 and 10 above)

Spine/back:

Please choose **all** that apply:

- ☐ Skull
- ☐ Neck
- ☐ Upper back
- ☐ Lower back
- ☐ Lameness not localized
- ☐ Other (describe):

16 JOINTS (if you checked Forelimb and Joint in question 8 and 10 above)

Forelimb:

Please choose **all** that apply:

- ☐ Shoulder
- ☐ Elbow
- ☐ Knee (carpus)
- ☐ Fetlock
- ☐ Pastern
- ☐ Coffin
- ☐ Lameness not localized
- ☐ Other (describe):

17 Forelimb fetlock specifics:

Please choose **all** that apply:

- ☐ P1 (proximal phalanx) chip
- ☐ PSB (proximal sesamoid bone) chip
- ☐ Other periarticular fracture
- ☐ Lateral condylar fracture
- ☐ Arthritis or arthrosis
- ☐ Palmer metacarpal disease
- ☐ Other (describe):

18 **<u>JOINTS (if you checked Hindlimb and Joint in question 8 and 10 above)</u>**

<u>Hindlimb:</u>

Please choose **all** that apply:

- ☐ Hip
- ☐ Stifle
- ☐ Hock (tarsus)
- ☐ Fetlock
- ☐ Pastern
- ☐ Coffin
- ☐ Lameness not localized
- ☐ Other (describe):

19 Hindlimb fetlock specifics:

Please choose **all** that apply:

- ☐ P1 (proximal phalanx) chip
- ☐ PSB (proximal sesamoid bone) chip
- ☐ Other periarticular fracture
- ☐ Lateral condylar fracture
- ☐ Arthritis or arthrosis
- ☐ Plantar metatarsus disease
- ☐ Other (describe):

20 **<u>JOINTS (if you checked Spine and Joint in question 8 and 10 above)</u>**

<u>Spine/back:</u>

Please choose **all** that apply:

- ☐ Sacroiliac
- ☐ Neck
- ☐ Lameness not localized
- ☐ Other (describe):

21 LIGAMENTS / TENDONS (if you checked Forelimb and Ligaments/Tendons in question 8 and 10 above)

Forelimb:

Please choose **all** that apply:

- ☐ Suspensory ligament
- ☐ Distal sesamoidean ligament
- ☐ Superficial digital flexor tendon
- ☐ Proximal check ligament (above carpus)
- ☐ Deep digital flexor tendon
- ☐ Distal check ligament (below carpus)
- ☐ Other (describe):

22 LIGAMENTS / TENDONS (if you checked Hindlimb and Ligaments/Tendons in question 8 and 10 above)

Hindlimb:

Please choose **all** that apply:

- ☐ Suspensory ligament
- ☐ Distal sesamoidean ligament
- ☐ Superficial digital flexor tendon
- ☐ Deep digital flexor tendon
- ☐ Other (describe):

23 Did you reach a definitive diagnosis?

Please choose **only one** of the following:

- ○ Yes
- ○ No

24 How did you reach the diagnosis and/or what diagnostic procedures did you use?

Check ALL that apply

	What procedures where used?	Which procedures provided the definitive diagnosis?
**Clinical evaluation**	☐	☐
**Diagnostic analgesia**	☐	☐
**Radiography**	☐	☐
**Nuclear scintigraphy**	☐	☐
**Ultrasonography**	☐	☐
**Diagnostic arthroscopy**	☐	☐
**Other**	☐	☐

25 If 'other', please describe:

26 Was the horse treated for its diagnosed condition(s)?

Please choose **only one** of the following:

○ Yes

○ No

27 What was the treatment?

Please choose **all** that apply:

- ☐ Lay-up
- ☐ Medications
- ☐ Surgery
- ☐ ESWT (shockwave)
- ☐ Hyperbaric
- ☐ Physical therapy
- ☐ Other treatment (describe):

28 What was the treatment or outcome for ligament and/or tendon treatments?

Please choose **only one** of the following:

- ○ No change
- ○ Worsened
- ○ Improved

29 Please list anything else you think is important or provide more detail about the lameness diagnostic procedures below:

Please provide more detailed information, especially where there were more than one lameness incidents diagnosed.

30 What was the outcome?

Check ALL that apply

Please choose **all** that apply:

- ☐ Remained in training (no disruption)
- ☐ Returned to racing with previous performance
- ☐ Returned to racing with lower performance
- ☐ Retired from racing
- ☐ Other treatment (describe):

Medication

31 Are you aware of any medications administered while the horse has been under your care or otherwise?

Please choose **only one** of the following:

- ○Yes
- ○No
- ○Unknown

INTRA-ARTICULAR OR INTRATHECAL (JOINT OR TENDON SHEATH) MEDICATION

32 For each time period, what medications were administered and/or what procedures were conducted via the joint or tendon sheath?

Check ALL that apply

	None	Unknown	Previous week	Previous month	> 1 month
**Joint block**	☐	☐	☐	☐	☐
**Corticosteriods**	☐	☐	☐	☐	☐
**PSGAGs**	☐	☐	☐	☐	☐
**Hyaluronic acid**	☐	☐	☐	☐	☐
**IRAP (Interleukin-1 Receptor Antagonist Protein)**	☐	☐	☐	☐	☐
**Antibiotic**	☐	☐	☐	☐	☐
**Stem cells**	☐	☐	☐	☐	☐
**Other**	☐	☐	☐	☐	☐

33 If 'other', please describe:

SYSTEMIC MEDICATION (PO, IV, IM)

34 For each time period, what medications were administered via PO, IV or IM?

Check ALL that apply

	None	Unknown	Previous week	Previous month	> 1 month
**Phenylbutazone (bute)**	☐	☐	☐	☐	☐
**Flunixin (Banamine)**	☐	☐	☐	☐	☐
**Ketoprofen**	☐	☐	☐	☐	☐
**Robaxin (muscle relaxant)**	☐	☐	☐	☐	☐
**Other**	☐	☐	☐	☐	☐

35 If 'other', please describe:

TENDON OR LIGAMENT INJECTIONS

36 For each time period, what medications were administered or procedures were conducted on the tendons or ligaments?

Check ALL that apply

	None	Unknown	Previous week	Previous month	> 1 month
**Diagnostic block**	☐	☐	☐	☐	☐
**Platelet rich plasma (PRP)**	☐	☐	☐	☐	☐
**IRAP (Interleukin-1 Receptor Antagonist Protein)**	☐	☐	☐	☐	☐
**Stem cells**	☐	☐	☐	☐	☐
**Other**	☐	☐	☐	☐	☐

37 If 'other', please describe:

38 Please list anything else you think is important or provide more detail about the medication or medication strategies below:

Please provide more detailed information on specific drugs administered, dosage and length of time on medications.

Surgery

39 Are you aware of any surgeries while the horse has been under your care or otherwise?

Please choose **only one** of the following:

- ◯ Yes
- ◯ No
- ◯ Unknown

40 What surgical procedures were performed in the designated time periods?

Check <u>ALL</u> that apply

	0 -3 months	3 - 6 months	6 - 12 months	> 12 months
**Arthroscopy**	☐	☐	☐	☐
**Other orthopedic w/ screw fixation**	☐	☐	☐	☐
**Other orthopedic w/ plate fixation**	☐	☐	☐	☐
**Non-orthopedic soft tissue surgery**	☐	☐	☐	☐
**Other**	☐	☐	☐	☐

41 If other, please describe:

42 What structure(s) were surgically treated in the designated time periods?

Check <u>ALL</u> that apply

	0 - 3 months	3- 6 months	6 - 12 months	> 12 months
**Forelimb fetlock**	☐	☐	☐	☐
**Hindlimb fetlock**	☐	☐	☐	☐
**Carpus**	☐	☐	☐	☐
**Tarsus**	☐	☐	☐	☐
**Forelimb splint bone resection**	☐	☐	☐	☐
**Hindlimb splint bone resection**	☐	☐	☐	☐
**Other**	☐	☐	☐	☐

43 Forelimb fetlock specifics:

Please choose **all** that apply:

- ☐ Lateral condylar fracture
- ☐ P1 (proximal phalanx) chip
- ☐ PSB (proximal sesamoid bone) chip
- ☐ Other periarticular fracture
- ☐ Other (describe):

44 Carpus specifics:

Please choose **all** that apply:

- ☐ Chip removal
- ☐ Slab fracture
- ☐ Other periarticular fracture
- ☐ Other (describe):

45 Hind fetlock specifics:

Please choose **all** that apply:

- ☐ Lateral condylar fracture
- ☐ P1 (proximal phalanx) chip
- ☐ PSB (proximal sesamoid bone) chip
- ☐ Other periarticular fracture
- ☐ Other (describe):

46 Tarsus specifics:

Please choose **all** that apply:

- ☐ Chip removal
- ☐ Slab fracture
- ☐ Other periarticular fracture
- ☐ Other (describe):

47 If other, please describe:

48 Please list anything else you think is important or provide more detail about the surgery or surgeries below:

Include more detailed information on limb, structure and surgery.

Thank you for completing this Equine Medical History Survey.
