# Supplementary material for: Relationship Between Historical Lameness, Medication Usage, Surgery, and Exercise With Catastrophic Musculoskeletal Injury in Racehorses
Source: Front Vet Sci. 2018 Sep 7;5:217. doi: 10.3389/fvets.2018.00217 (PMC6137211; doi:10.3389/fvets.2018.00217)
Supplement: Supplementary Table 1 — Affected structures of racehorses with poor performance or lameness problems in the months prior to case death, stratified by racehorse breed. [file Table_1.DOCX]

**Supplementary Table 1.** Affected structures of racehorses with poor performance or unsoundness problems in the months prior to case death, stratified by racehorse breed

|  | **TB** | | **QH** | |
| --- | --- | --- | --- | --- |
|  | **Cases**  **(n=42)** | **Control**  **(n=95)** | **Case**  **(n=11)** | **Control**  **(n=6)** |
| Unsoundness | 21 | 42 | 2 | 2 |
| Localized to structure | 20 | 38 | 2 | 2 |
| Left forelimb |  |  |  |  |
| 0-3 months | 4 | 6 | 0 | 0 |
| 3-6 months | 4 | 6 | 0 | 1 |
| 6-12 months | 2 | 9 | 1 | 0 |
| > 12 months | 2 | 4 | 0 | 0 |
| Right forelimb |  |  |  |  |
| 0-3 months | 6 | 7 | 1 | 1 |
| 3-6 months | 3 | 5 | 0 | 0 |
| 6-12 months | 2 | 7 | 1 | 0 |
| > 12 months | 3 | 4 | 0 | 0 |
| Left hindlimb |  |  |  |  |
| 0-3 months | 1 | 5 | 0 | 1 |
| 3-6 months | 0 | 1 | 0 | 0 |
| 6-12 months | 1 | 2 | 1 | 0 |
| > 12 months | 3 | 3 | 0 | 0 |
| Right hindlimb |  |  |  |  |
| 0-3 months | 2 | 5 | 0 | 0 |
| 3-6 months | 0 | 1 | 0 | 0 |
| 6-12 months | 1 | 5 | 1 | 0 |
| > 12 months | 2 | 1 | 0 | 0 |
